# Global systematic review of occupational health and safety outcomes among sanitation and hygiene workers

**DOI:** 10.3389/fpubh.2023.1304977

**Published:** 2023-12-19

**Authors:** Sina Temesgen Tolera, Shibiru Temesgen, Salie Mulat Endalew, Tamagnu Sintie Alamirew, Liku Muche Temesgen

**Affiliations:** ^1^College of Health and Medical Sciences, Haramaya University, Dire Dawa, Ethiopia; ^2^Department of Statistics, Addis Ababa University, Addis Ababa, Ethiopia

**Keywords:** global, health, occupation, outcomes, safety, sanitary workers

## Abstract

**Introduction:**

Sanitary workers are exposed to a variety of occupational hazards in a variety of working environments, which can result in occupational-related outcomes. As a result, the goal of this review was to identify occupational health and safety outcomes among sanitary workers worldwide.

**Methods:**

PRISMA was used as flow diagram and PICOS was used review questions. The studies published in English were searched from databases and others methods ranging from 2000 to 2022. Boolean logic (AND, OR), MeSH, and keywords used: (Occupation *OR Job *OR Work) AND (Occupational related respiratory Symptoms *OR Disease) AND [Solid waste collectors (SWCs) *OR Street sweepers (SS) *OR Sewage workers and waste treatment (STWs)] AND (Countries).

**Results:**

A total of 228 studies were identified from 23 countries across the world. Studies were found via PubMed (*n* = 40), Medline (*n* = 25), Embase (*n* = 11) and Global Health (*n* = 66) and Google scholar (*n* = 63) and from previous (*n* = 23). From 8,962 of eligible sanitary workers, about 4,742 (54%), 1714 (19%) and 1,441 (16%) were sewage, sweepers and solid waste workers, respectively. A total study (*n* = 51) were eligible for occupational health and safety outcomes. Of these, respiratory problems accounted 27 (52%) and Gastroenteritis 14 (27%).

**Conclusion:**

Despite a large number of studies to date provides sanitary employees all over the world face occupational-related risks, hence more research is needed to enhance and quantify illness burden among sanitary workers.

## Introduction

Sanitary workers are those who clean health facilities, latrines, toilets, pits, offices, sewers, sewage treatment, manholes, sweeping streets, waste collection, fecal management, and handling sludge (1–3) and are essential to global public health and societal wellbeing (4, 5). However, due to poor occupational health and safety practices, these groups are exposed to excreted bodily fluids, blood, and infectious waste material suspected to contain pathogens (bacteria, viruses, parasites, or fungi); infectious agent cultures and stocks from laboratory work; and waste from infected patients in isolation wards (6–8). Moreover, the other study found that they are facing cuts, injuries, hepatitis A, hepatitis B, hepatitis C virus, and other occupational-related diseases (9). Such injuries and illnesses affect the job performance of the cleaners, thus affecting their efficiency. Due to reduced efficiency and absenteeism, they have to incur losses in wages, and the treatment and rehabilitation of these employees are costly to society (10).

As the result, WHO reports, millions of sanitation workers in the developing world are forced to work in conditions that endanger their health and lives, and violate their dignity and human rights (2). They are often the most marginalized groups, discriminated against by members of society, carrying out their jobs with no equipment and no legal rights (2); poor in terms of economy (11); paying little attention to OHS, and socially stigmatized (2). Beside these, they are often neglected with challenges of insecurity in financial status and social issues, like social stigma like intergenerational discrimination (9, 12). Moreover, the tasks performed by cleaners are labor-intensive, and most of the cleaners have to work under time constraints, increasing their physical and mental stress (10).

Now-day, increasing population in Africa, Asia, and South America and the attention given to sanitary workers are mismatched (13). For example, the study found in India indicated that of the 5 million sanitary workers, more than 2.5 million were exposed to various occupational hazards in service-giving industries, but not as sounded to report them to concerned bodies (14). Also, sanitary workers are facing psychological and mental problems with the intensity of work (15); job insecurity, and acts of job violence arising out of or in connection with work (16). As a result, they were dissatisfied with their daily work activities (17).

Occupational outcomes are a common cause of morbidity, disability, and poor quality of life, which range from 56 to 90%. Occupational outcomes are the consequences of occupational hazards, which might be occupational-related diseases, injuries, or musculoskeletal disorders. The rate of occupational injuries and illnesses among sanitary workers was 3.9 per 100 full-time workers (10). However, compiled information on occupational health and safety outcomes among sanitation workers is neither well understood nor well quantified, particularly in developing countries. Therefore, it is important to conduct a systematic review that could inform the production of a global burden of occupational-related diseases or disabilities for further evidence. As a result, the overarching goal of this systematic review was to identify occupational health and safety outcomes for sanitary workers worldwide: cross-sectional Research.

## Method

### Review protocols

The flow diagram for the Preferred Reporting Items for Systematic Reviews (PRISMA) updated protocol was used (18). For systematic review questions, the PICOS (Population, Intervention, Comparison, Outcome, and Study Type) protocol was used.

### Study eligibility criteria

#### Inclusion criteria

- *Population* stands for sanitary workers, namely solid waste collectors, health care facility cleaners, sewage workers, waste water treatment workers, and sweeping streets working-age population.
- *Intervention:* Occupational-related exposure
- *Comparison:* Not applicable because the review only focuses on a descriptive cross-sectional study.
- *Outcome*: OHS-related outcomes include respiratory track diseases, gastroenteritis, and mental and social health conditions.
- *Study type*: An observational study (cross-sectional study) only included.
- *Language:* All studies published in English
- *Articles/Studies:* Articles with their full texts and abstracts available in English with clear objectives and methodology, studies, and quantitative outcomes included
- *Publication Year:* From Year of DD/MM/YY: 1/1/2000–2022

#### Exclusion criteria

- *Population:* office cleaners, hotel and restaurant cleaners were excluded from this review
- *Study Design:* Non-cross-sectional studies like Randomized controlled trials (RCTs) that are individually-or cluster-RCT and the following non-randomized controlled studies (NRS): quasi-RCTs, non-RCTs, controlled before and-after studies, historically controlled studies, interrupted-time-series studies, case–control studies and cohort studies.
- *Language:* Studies published in non-English languages
- *Articles/Studies:* studies that do not have clear objective and methodology; studies excluded
- *Publication Year:* Studies prior to 1/1/2000 years were not included in this review

### Searched engines/databases

Systematical Review was searched from database namely PubMed, MEDLINE, Embase, Global Health electronic databases and other searches like Google scholar and home pages.

### Searching strategies

The studies published from 2000 to 2022 were identified through PubMed, Medline, Embase, and Global Health electronic databases using EndNote online searches and from others. The keywords and MeSH terms were used as Boolean logic operators (AND” or “OR”) individually or in conjunction as: (Occupational *OR Job *OR Work) **AND** (Diseases*OR Gastroenteritis or Respiratory *OR Mental Health Condition *OR Health Problems occupational*OR work place) **AND** (Sanitary Workers *OR Street sweepers *OR Solid Waste Collectors *OR Sewage Workers *OR Waste Treatment Workers) **AND** Countries (Developing and Developed Countries).

### Data screening

Three reviewers screened titles and abstracts and full text using Microsoft Excel, and full copies of titles and abstracts were obtained. Then finally, the results from the databases were managed and removed in the reference management EndNote 9.2 and Zotero, respectively.

### Data extraction

There were three reviewers on this job. A prescribed extraction form created in a Microsoft Excel spreadsheet was used to extract data. It includes main outcomes, authors with year, country, and job categories, an outcome assessment tool, and a quality evaluation tool.

### Data synthesis

Two reviewers were involved in this task. The studies published pertaining to occupational health and safety outcomes were tabulated, described, and synthesized according to the type of outcomes.

### Quality assessment

Two reviewers assessed all published studies using the Joanna Briggs Institute (JBI) Critical Appraisal Checklist, which was adapted (19). It has nine criteria that emphasize: (1) an appropriate sample frame to address the target population; (2) an appropriate way of sampling study participants; (3) an adequate sample size; (4) a description of both study subjects and the setting; (5) data analysis with a sufficient sample; (6) valid methods used for identification; and (7) conditions measured in a standard, reliable way for all participants. (8) Statistical analysis appropriateness: (9) Appropriate response rate All of these were scored as (1) Yes, (2) No, (3) Uncertain, and (4) Not applicable. Finally, if the article received less than five points out of nine “yes,” it indicates a high publication risk or low paper quality; 5–7 indicates a medium publication risk; and 8–9 indicates a low publication bias.

## Results

### Selection studies

**Figure 1:**
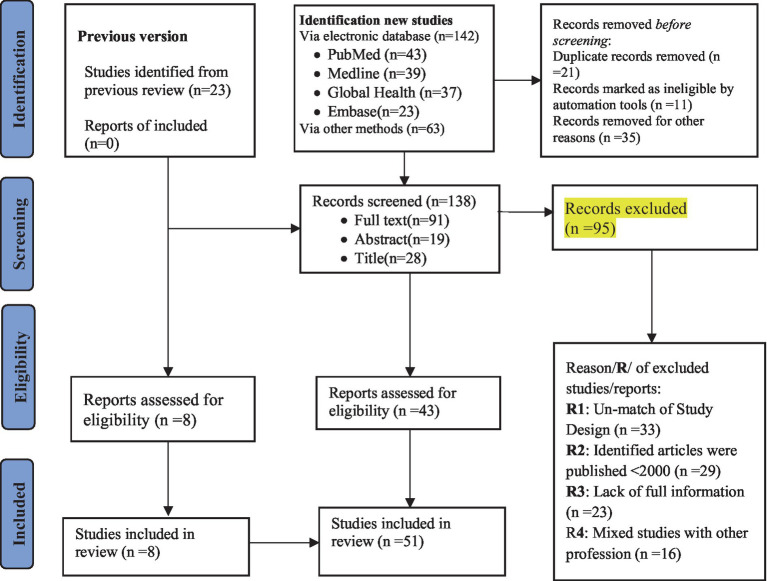
Flow diagram for systematic reviews adopted from PRISMA 2020.

A total of 228 studies were identified from the databases and other retrieved data and reports. Of these, 23 studies were from studies included in the previous version of the review, 142 studies were from new studies via databases, and 63 studies were from new studies via other methods. Finally, a total of 51 studies were included in this systematic review (Figure 1).

### Study overview

Fifty-one eligible studies were presented in Table 1, which has rows for authors, countries, study design, tool used for assessment, number of sanitary workers with their categories, outcomes, and article quality/publication bias (Table 1).

**Table 1:** Eligible studies included in the review health outcomes, population and assessment.

Outcomes	Authors	Country	Design	Tool used for assessment	Study population (*n* = 8,962)	Identified outcomes	Publ. bias
Occupational related respiratory diseases (*n* = 27)	Chandra et al. (20)	India	CS	Questionnaires	Sewage workers (*n* = 104)	Pulmonary TB; COPD; asthma	Low
	Cyprowski et al. (21)	India	CS/CG	Questionnaires, lab. test	Street sweepers (*n* = 50)	Occupation related lung diseases	Low
	Cyprowski et al. (22)	Poland	CS	Spirometer measurement	Sewage workers (*n* = 78)	Lung function	Medium
	El-Hamid et al. (23)	Poland	CS	Questionnaire	Sewage workers (*n* = 38)	Inflammatory mediators; interleukin CONC.	Low
	Heldal et al. (24)	Egypt	CS	Questionnaires, spirometer	Sanitary workers (*n* = 21)	Bronchial hyper-responsiveness	Medium
	Heldal et al. (25)	Egypt	CS	Questionnaire, obse. and lab test	Sewage workers (*n* = 140)	Sewage workers had unhealthy appearance	Medium
	Heldal et al. (26)	Norway	CS	Questionnaires, spirometer	Sewage workers (*n* = 44)	Lung function and health symptoms	Low
	Shadab et al. (27)	Norway	CS	PAS 6 cassettes and PS101	Sewage workers (*n* = 82)	Serum-levels of pneumoproteins-CC16,SP-A& SP-D	Medium
	Ajay et al. (28)	Norway	CS	Blood sampling, spirometer	Waste water workers (*n* = 148)	Inflammatory effects; lung function	Medium
	Anwar et al. (29)	India	CS	Spiro lab II spirometer	Sewage workers (*n* = 62)	Pulmonary, oxidative stress	Low
	Arora et al. (30)	Pakistan	CS/CG	Questionnaires, spirometer	Street sweepers (*n* = 100)	Impairs lung function	Low
	Erah et al. (31)	India	CS/CG	Spirometer	Street sweepers (*n* = 120)	Lung function problems	Low
	Johncy et al. (32)	Nigeria	CS	Questionnaires	Street sweepers (*n* = 46)	Cough, phlegm, chest pain, noisy breathing, sneezing	High
	Stambuli (33)	India	CS/CG	Spirometer	Street sweepers (*n* = 60)	Impact of dust on lung functions	Low
	Shadab et al. (34)	India	CS/CG	Questionnaires	Street sweepers (*n* = 120)	Nose irritation, sneezing, rhinitis, cough, phlegm, wheezing	Medium
	Eshaghi Sani (35)	India	CS/CG	Spirometer	Street sweepers (*n* = 60)	Decreased lung function	Low
	Sangolli et al. (36)	India	CS/CG	Interview and spirometer	Street sweepers (*n* = 86)	Lung impairment	Low
	Eneyew et al. (37)	Tanzania	CS	Questionnaires	Street sweepers (*n* = 102)	cough, phlegm, sneezing, nose irritating, wheezing	Low
	Mostafa et al. (38)	India	CS	Spirometer	Street sweepers (*n* = 110)	COPD pattern of impaired lung functions	Low
	Nku et al. (39)	Iran	CS	Spirometer	Street sweepers (*n* = 100)	Lung problems	Low
	Johncy et al. (40)	India	CS	Interview and spirometer	Street sweepers (*n* = 80)	Cough, chest pain, catarrah, and sneezing	Medium
	Johncy et al. (41)	Ethiopia	CS	Questionnaires, Obse. checklist	Waste collectors (*n* = 546)	Cough, wheezing, phlegm, chest illness, and breath	Low
	Juhi (42)	Ethiopia	CS	Questionnaires	Street sweeper, SWC (*n* = 168)	Acute respiratory Infection	Medium
	Emiru et al. (43)	Egypt	CS/CG	Questionnaires, spirometer	Street sweepers (*n* = 207)	Pulmonary problems	Low
	Singh and Ladusingh (44)	Nigeria	CS	Pulse dosimeter	Street sweepers (*n* = 200)	impairs lung function, cough, chest, sneezing	Low
	Athanasiou et al. (45)	India	CS	Questionnaires	SWC (*n* = 224); sewage workers (*n* = 51)	Chronic bronchitis	Medium
	Fahim and El-Prince (46).	Greece	CS	Questionnaires, spirometer	SWC (*n* = 104)	Breathlessness, phlegm, cough, wheezing	Low
Occupational related gastroenteritis (*n* = 14)	Bonanni et al. (47)	Italy	CS	Serological analysis	Sewage workers (*n* = 225)	Hepatitis A Virus	Medium
	Divizia et al. (48)	Italy	CS	questionnaire, blood sample	Sewage workers (*n* = 138)	Sero-positivity to HAV, echovirus types 1 and 9	Low
	Levin et al. (49)	Egypt	CS	HEV IgG detection,	Sewage workers (*n* = 205)	Hepatitis E Virus	Medium
	Montuori et al. (50)	Uganda	CS	Stool and wastewater samples used	Sewage workers (*n* = 231)	Intestinal parasites; soil- helminthes	Low
	Toseva et al. (51)	Egypt	CS	Stool sample	Sewage workers (*n* = 410)	*H. pylori* infection and viral hepatitis	Medium
	El-Esnawy et al. (52)	Malaysia	CS	Microscopic agglutination	Sanitary workers (*n* = 303)	Leptospirosis	Low
	Venczel et al. (53)	Israel	CS	Serological analysis	Sewage workers (*n* = 100)	Sero positivity to Hepatitis A	Low
	Hassanein et al. (54)	Italy	CS	Blood serology	Wastewater workers (*n* = 869)	Hepatitis A virus	Medium
	VanHooste et al. (55)	Austria	CS	Stool sample	Sewage workers (*n* = 46)	*Tropheryma whipplei*	Medium
	Jeffree et al. (56)	Bulgaria	CS	Blood sample	Wastewater workers (*n* = 110)	Anti-HAV Antibodies	Medium
	Fuhrimann et al. (57)	India	CS	Blood sample	Sewage workers (*n* = 147)	Anti-HEV-IgGAntibodies	Low
	Schöniger-Hekele et al. (58)	Belgium	CS	Blood sample	Sewage workers (*n* = 317)	Helico bacter pylori infections; GI symptoms	Low
	Thorn and Beijer (59)	USA	CS	Blood sample	Sewage workers (*n* = 365)	Hepatitis E virus	Medium
	Thorn et al. (60)	Sweden	CS	Questionnaire, spirometer	Sewage workers (*n* = 114)	Symptoms in gastrointestinal	Medium
Mental and social (*n* = 1)	Lee et al. (61)	India	CS	Questionnaires, checklist	Sewage workers (150)	Occupational stress	Medium
Gastroenteritis and respiratory (*n* = 4)	Sangkham et al. (62)	Netherlands	CS	Questionnaires, endotoxin measure	Sewage workers (*n* = 151)	RT, Irritation, neurological, GI	Low
	Lenka (63)	Poland	CS	Questionnaires, endotoxin	Sewage workers (*n* = 99)	General health symptoms	Low
	Preisser et al. (64)	Germany	CS	Spirometer	Street sweepers, waste Collectors (*n* = 61)	The, obstructive lung disease (FEV1/ FVC)	Medium
	Uhunamure et al. (65)	Netherlands	CS	Endotoxin measurement	Wastewater workers (*n* = 99)	Respiratory and GI symptoms	Medium
Gastroenteritis, respiratory, mental and social, MSDs, skin conditions (*n* = 7)	Douwes et al. (66)	Sweden	CS	Questionnaires	Sewage workers (*n* = 257)	Work-related symptoms	Low
	Krajewski et al. (67)	United States	CS	Endotoxin measurement	Wastewater workers (*n* = 91)	RT, GI, ocular and skin irritations, and neurology	High
	Smit et al. (68)	India	CS/CG	Pretested p	SS (*n* = 273)	Health problems	Medium
	El-Wahab et al.(69)	South Africa	CS	Questionnaires, Obse. checklist	Waste Collectors (*n* = 114)	Skin, GI, RT and MSDs, eye, mental health, skin	Low
	Giri et al. (70)	Thailand	CS	Questionnaires, Obse. Overall quality	Waste Collectors (*n* = 107)	Occupational injuries, MSDs, RT, GI, head, eyes, ears, and skin	Medium
	Lenka (71)	India	CS	Questionnaires, checklist	Sanitary workers (*n* = 110)	Cardiovascular degradation, skin rash, and RT	Medium
	El-Wahab et al. (72)	Egypt	CS	Questionnaires, Obse.	SWCs (*n* = 346)	GI, RI, skin, and MSDs.	Medium

CS, cross sectional study; CG, group control; FDG, focus group discussion; RI, respiratory infections GI, gastroenteritis; MSDs, musculoskeletal disorders; COPD, chronic obstructive pulmonary diseases; Obse, observational checklist; SCWs, solid waste collectors; SS, street sweepers.

### Eligible countries

**Figure 2:**
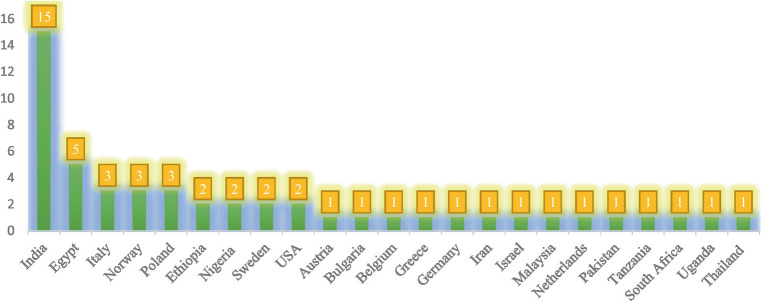
Number of studies identified by countries across the world wide.

A total of 51 studies from 23 different countries were reviewed. 14 of these countries were from developed countries, while 9 were from developing countries. India was the first leading developing country where the majority of the studies were discovered (Figure 2).

### Studied population

**Figure 3:**
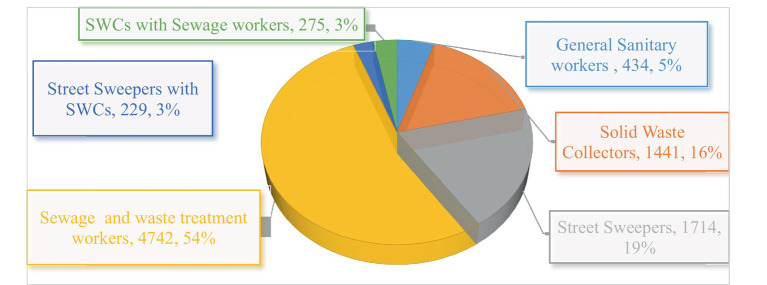
Categories of sanitary workers exposed to OHS problems reviewed 2000–2022.

From the total population (*n* = 8,962), 54% (4742) were sewage and waste treatment workers, followed by 1714 (19%) street sweepers and 1,441 (16%) municipal solid waste collectors. The remaining 434 (5%), SWCs with Sewage workers 275 (3.3), and street sweepers with SWCs 229 (2.7%) were general sanitary workers (Figure 3).

### Tools used for assessment

The majority of the studies used cross-sectional designs with structured, standard questionnaires alone, or questionnaires with observational checklists. A few of them used spirometer questionnaires, blood tests, and stool examinations (Supplementary Figure S2).

### Statistical technique

Statistically, nearly half of the researchers used logistic regression, binary and multivariate regression analysis, with chi-square 8 (16%) coming in second (Supplementary Figure S2).

### Publication bias

Fifty-one studies included in the review are presented in the following table and were evaluated based on JBI criteria, which have nine statements. From all these eligible studies, 459 points (each study evaluated by 9 statements) were expected, but only about 349/459 (76%) fulfilled the JBI criteria (Supplementary Table S1).

### Identified OHS outcomes

**Figure 4:**
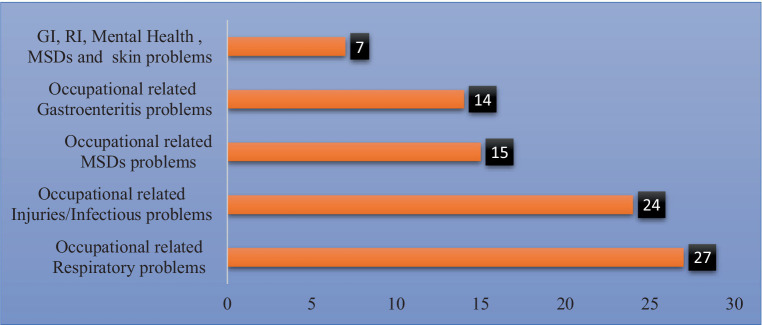
Numbers of studies identified on occupational health and safety outcomes, 2022.

Out of 51 studies, the majority, i.e., 27 (52%), focused on occupational-related respiratory problems. The remaining 14 (27%), 6 (11%), 4 (8%), and 1 (2%) of them focused on occupational-related combinations of GI, RT, and mental health conditions; GI and RT problems; and mental and social conditions, respectively (Figure 4).

## Discussion

The systematic review literature search yielded a total of two hundred twenty-eight studies from the databases and other retrieved data and reports. Of these, twenty-three studies were from studies included in the previous version of the review, one hundred forty-two studies were from new studies via databases, and sixty-three studies were from new studies via other methods. Then, one hundred thirty-eight studies were available for review after the removal of duplicate records. Seventy studies were excluded due to unmatched design, publication before 2000 years, languages, and population type. Moreover, some reports were excluded due to the mixed study population with other occupations, lack of full information, and unclear methods and output (Figure 1). After title and abstract screening, forty-three studies were obtained from the new database using another method, while eight studies were obtained from previous studies.

As a result, a total of 51 studies were considered potentially eligible for inclusion in this systematic review. Thus, a large number of studies met the review’s inclusion criteria, representing a potentially large body of evidence for this review. Eligible studies were arranged by authors with date of publication, countries, study design, categories of sanitary workers, types of outcomes, and outcome assessment tools across the world (Table 1). These studies and articles were identified in 32 countries. Of these countries, 14 (61%) were from developed countries, and 9 (39%) were from developing countries (Figure 2). However, more than half of the data was extracted from articles published by developing countries. Of these countries, the majority of studies were obtained from India (*n* = 15), followed by Egypt (*n* = 5). Nine studies were obtained from Poland (*n* = 3), Norway (*n* = 3), Italy (*n* = 3), and Ethiopia, Nigeria, the Netherlands, the United States, and Sweden (from each, *n* = 2 studies). The rest of the thirteen studies (13) were from Austria, Belgium, Bulgaria, Germany, Greece, Iran, Israel, Malaysia, Pakistan, South Africa, Tanzania, Uganda, and Thailand (from each *n* = 1 study) (Figure 2).

Pertaining to the study, population, health care facility cleaners, general sanitary workers, municipality solid waste collectors (SWCs), sewage and waste treatment workers, street sweepers, and waste collectors were included. Out of a total of eight thousand nine hundred sixty-two sanitary workers, more than half were sewage and waste treatment workers. The rest were waste treatment workers, street sweepers, SWCs, sanitary workers, SWCs with sewage workers, and street sweepers with SWCs, in decreasing order (Figure 3).

In terms of study design and tools, 51 studies used questionnaires with an observational checklist. Nine studies used questionnaires with spirometers, and two studies used questionnaires with endotoxin measurements. Moreover, eight studies demonstrated spirometer measurement to detect respiratory problems among sanitary workers. For outcome assessment, laboratory confirmation and/or physician diagnoses were used in most studies; other studies relied on personal recall. Some of the laboratory and/or physician assessments consisted of analyses of bio-samples i.e., blood sample (*n* = 7), stool sampling (*n* = 3), blood sampling with a spirometer (*n* = 1), endotoxin measurement (*n* = 2), PAS 6 cassettes and PS101 (*n* = 1), pulse dosimeter (*n* = 1), and microscopic agglutination (*n* = 1) (Supplementary Figure S1). As Table 1 shows, stool examination is demonstrated for gastroenteritis to detect the presence of microbial, intestinal parasite infections, and hepatitis A and B viruses in sanitary workers, while spirometer measurement is used for respiratory examination (Table 1). Besides, in sewage workers, waste water was analyzed to know the load of bacteriology in sewage water and waste treatment, either risky or not, depending on the possibilities of exposure (Table 1). Statistically, the majority of the studies used logistic regression, binary and multiple, bivariate, and multivariate regression analyses. Followed by chi-square with other models such as chi-square with Fisher’s exact test, logistic regression, multiple comparisons, and binary logistic regression (Supplementary Figure S2).

Regarding the quality of the paper and publication bias, all included studies were evaluated based on JBI criteria, which have nine statements for cross-sectional studies. As a summary of this issue, for 51 studies, it was expected that 459 points would fulfill the JBI criteria, but only about 349/459 (76%) fulfilled the JBI criteria (Supplementary Table S1). In this case, selection bias was common. Of these, the study participants were sampled in an appropriate sample frame to address the target population and the problem of valid methods used for the identification of the condition. Most studies did not specify the inclusion/exclusion criteria or selection method for workplace sanitation workers. When it comes to occupational health and safety outcomes, a large range of studies were identified in terms of studies focused on occupational-related respiratory diseases, occupational injuries, musculoskeletal disorders, gastroenteritis, and mental and social conditions. Each of these outcomes will be presented separately (Figure 4).

*Occupational-related respiratory diseases:* respiratory problems were the first most common occupational-related outcome among sanitary workers, with a total of 27 studies reporting on a variety of respiratory endpoints. Thus, the first largest outcome coverage of OHS outcomes is occupational-related respiratory diseases linked with sanitary workers. The majority of the assessment tools were questionnaires and Spiro-meter measurements. Eight studies from different countries found that sewage workers had common pulmonary TB, chronic obstructive pulmonary disease, bronchial asthma, impaired lung function, problems with inflammatory mediators, disruptions of pulmonary function, and were faced with oxidative stress (20–27).

Moreover, 15 studies revealed that street sweepers developed loss of lung elastic recoil pressure (28); impaired lung function (29, 30); cough, phlegm, chest pain, sneezing, noisy breathing, nose irritation, rhinitis, cough, and wheezing (31, 32). They developed an obstructive pattern of impaired lung functions and acute respiratory infection (33–39). The respiratory problems observed among street sweepers were due to the exposure of dust to lung functions (40) which leads to decreased lung function due to dust exposure (41); and lung impairment (42). Three studies conducted on solid waste collectors showed coughing, wheezing, phlegm, chest illness and breath problems (43), chronic bronchitis (44), and breathlessness, phlegm, coughing, and wheezing (45). From 27 studies, only one study conducted on general sanitary workers showed that they developed bronchial hyper responsiveness (46). As evidenced above, sanitary workers working as street sweepers, sewage workers, or municipal waste collectors have the possibility of developing respiratory problems if occupational safety materials aren’t well practiced.

*Occupational-related gastroenteritis:* Fourteen (14) studies pertaining to occupational-related gastroenteritis were identified across the world. From these studies, 13 studies were conducted on liquid waste management workers, sewage workers, and waste treatment workers. Occupational-related gastrointestinal conditions included symptoms of gastroenteritis (diarrhea, nausea, or stomach pain) or the presence of infectious agents in stool. As indicated above, the majority of sanitary workers under these conditions were sewage workers. The findings obtained from numerous studies indicated that sewage workers developed the hepatitis A virus (47–51). Moreover, this group of sanitary workers was exposed to the hepatitis E virus (52, 53); hepatitis B and C virus (54). Furthermore, waste water treatment workers were exposed to different microbial and protozoan infections. As this study indicated, microbial infections among sewage workers were 70.5%, followed by protozoan infections at 54.6, and 5.9% of them had helminthic infections. (54). In addition, they had the possibility of having *Helicobacter pylori* infections (55); leptospirosis (56); intestinal parasites; soil-based transmitted helminthes (57); arthralgia as the most prominent symptom (58); and also GI problems (59). The majority of these studies concluded that sanitary workers who had food and drink while they were working and who did not have personal protective equipment had the possibility of having gastroenteritis problems.

*Multiple occupational-related problems:* Seven (7) studies emphasized the combination of occupational-related respiratory, gastroenteritis, mental and social conditions, MSDs, and dermatology/skin conditions. Sanitary workers could develop multiple diseases while they are working. A study revealed that an increased risk for upper and lower airway effects such as nose irritation, congested nose, cough, breathlessness, wheezing, chest tightness, chronic bronchitis, and toxic pneumonitis was identified among sewage workers (60). Moreover, an increased risk for non-specific work-related gastrointestinal symptoms was found among the sewage workers; an increased risk for joint pains, related to pains in more than four joints among them (60). The other study showed that respiratory, ocular, and skin irritation, neurology, and gastro-intestinal symptoms were observed among waste treatment workers (61, 62). While cardiovascular degradation, MSDs, infections, skin problems, and RT problems found in sanitary workers (63). Moreover, health problems like hypertension, angina pectoris, myocardial infarction, chronic ischemic heart disease, heart failure, stroke, hemorrhoids, mono-neuropathy of the upper extremities, damage of the knee joint, back pain, synovitis and tenosynovitis, and other diseases of tendons and shoulder lesions were observed among street and SWCs (64). The other study conducted on waste collectors showed that dermatology or skin problems (10.53%), gastro-intestinal problems (7%), respiratory conditions (14.04%), musculoskeletal disorders (14.04%), eye problems (12.28%), and mental (21.05%) (65). Moreover, the prevalence of occupational injuries was 72.0%; musculoskeletal disorders (59.7%), respiratory symptoms (23.4%), head, eyes, and ears (7.8%), skin (5.2%), and gastrointestinal (3.9%) were common among waste collectors (62). Moreover, as in other studies conducted on street sweepers, they faced anemia (20.5%), hypertension (9.5%), upper respiratory tract infections (7.3%), and chronic bronchitis (5.9%).

*Occupational-related GI and TR:* Four (4) studies were identified on the issues of occupational-related gastroenteritis and respiratory tract problems. Of the four studies, three focused on sewage workers. As a result of their findings, they developed respiratory symptoms and gastrointestinal symptoms (nausea, acid indigestion, lack of appetite, vomiting during work, and diarrhea). Moreover, the study identified that they had irritation symptoms (runny nose, throat, skin, and eye irritations, and skin rash); neurological symptoms (headache, difficulty concentrating, forgetfulness, and dizziness); flu-like symptoms like fatigue, fever, shivering, perspiration, joint and muscle aches, and trembling limbs; and other symptoms like palpitation (66–68). Other studies also found in Egypt revealed that sewage workers had gastrointestinal (GIT) complaints such as abdominal colic’s (25.5%), diarrhea (24.5%), dyspepsia (24.3%), vomiting (10.9%), and dysentery (10.9%) (69). Almost all studies were found in developed countries. These show that the findings were the output of bio-sample and Spiro metric measurement, which are very easy to demonstrate in this world but difficult to apply in developing countries due to a lack of experts and the availability of these instruments.

*Mental health and social conditions:* Only one study of the mental and social health conditions of sanitary workers found that nearly 66.67% of them had moderate to high occupational-related stress. The majority (77.33%) of the workers worked for more than 10 years. As per this report, 99.33% of them were powerless, 84.00% of them were due to strenuous working conditions and unprofitability, and 74.00% of them were due to intrinsic impoverishment as the predominant sub-scales in the high occupational stress index. The study also addressed alcohol addiction; 66% of workers with low stress, 65% of workers with moderate stress, and 80% of workers with high stress responded that they were addicted to alcohol. As this was reported, it was predicted that socio-demographic factors influenced the occupational stress index. For example, the severity of occupational-related stress levels decreased with an increase in education status. Moreover, as reported, occupational-related stress increased as the duration of service increased (70).

### Limitations

Heterogeneity among the studies’ setting, population, study design, exposure assessment, and outcome assessments was observed. Moreover, the search did not independently assess publication bias, though that has been shown to be present in other reviews of sanitation interventions. Furthermore, many articles did not define their studies’ sanitation worker population of interest or may not have specified that their sanitation workers were exposed to human fecal sludge or wastewater, solid waste, or hospital hazardous waste, which inadvertently excluded them from the review. Regarding the use of this evidence for informing official global norms and standards, this systematic review demonstrates that more and better primary studies from a more diverse set of regions and countries are required to arrive at a body of evidence that would allow producers of official statistics to consider quantifying the work-related burden of disease and injury among sanitation workers. On the other side, almost all of the included studies used a cross-sectional study design, which might create selection bias and information bias at the sampling stage, and confounders might be one of the weak areas of this review. Therefore, the extent to which existing research can form a reasonable basis for policy or even estimates of the burden of disease is very limited due to the gaps in research and scientific rigor.

## Conclusion

Despite the limitations, the consistency of the evidence suggests that whatever sanitation workers are working in, they are facing occupational-related diseases like respiratory conditions, other occupational-related diseases, gastroenteritis, or mental or social health conditions. Moreover, this review demonstrates a clear need for further quantification of occupational health risks faced by sanitation workers to amend the effectiveness of governmental policies and other efforts to mitigate these risks across the world, particularly in low-income countries. Thus, more research is needed to improve the current bodies of evidence for all included health outcomes to be able to quantify disease burden among sanitary workers.

## Data availability statement

The original contributions presented in the study are included in the article/Supplementary material, further inquiries can be directed to the corresponding author/s.

## Author contributions

SiT: Writing – review & editing, Writing – original draft. ShT: Writing – original draft, Writing – review & editing. SM: Writing – review & editing. TA: Writing – review & editing. LT: Supervision, Writing – review & editing.

## References

[1] Kabir A Nadia F Farzana A Shahana J Ahsan A. Sweeping practices, knowledge about OSH hazards in Dhaka city, Bangladesh:. Aa qualitative inquiry, (2015). 2: 237–243.

[2] WHO . New report exposes horror of working conditions for millions of sanitation workers in the developing world. (2019). Available at: https://www.who.int/news/item/14-11-2019-new-report-exposes-horror-of-working-conditions-for-millions-of-sanitation-workers-in-the-developing-world.

[3] Gomathi P Kamala K. Threatening health impacts and challenging life of sanitary workers. J Evolution Med Dent Sci. (2020) 9:3055–61. doi: 10.14260/jemds/2020/669

[4] Bazzano A Oberhelman R Potts K Gordon A Var C. Environmental factors and WASH practices in the perinatal period in Cambodia. Int J Env Res Public Health. (2015) 12:2392–410. doi: 10.3390/ijerph120302392 25711360 PMC4377908

[5] KARJALAINEN A KURPPA K MARTIKAINEN R KLAUKKA T KARJALAINEN J. Work is related to a substantial portion of adult-onset asthma incidence in the Finnish population. Am J Respir Crit Care Med. (2001) 164:565–8. doi: 10.1164/ajrccm.164.4.2012146, PMID: 11520716

[6] Dement J Epling C Østbye T Pompeii LA Hunt DL. Blood and body fluid exposure risks among health care workers: results from the Duke health and safety surveillance system. Am J Ind Med. (2004) 46:637–48. doi: 10.1002/ajim.20106, PMID: 15551378

[7] Talaat M Kandeel A el-Shoubary W Bodenschatz C Khairy I Oun S . Occupational exposure to needle stick injuries and hepatitis B vaccination in Egypt. Am J Infect Control. (2003) 31:469–74. doi: 10.1016/j.ajic.2003.03.003, PMID: 14647109

[8] WHO . Safe management of wastes from health-care activities manual. (2014). (2nd Edn.). 1–24. Available at: https://iris.who.int/handle/10665/85349.

[9] Sperandeo L Srinivasan S. The heroes behind sanitation - an insight into faecal sludge management workers in Zambia. Zambia: BORDA (2020).

[10] BLS . Incidence rate of nonfatal occupational injuries and illnesses by industry and case types. United States Department of Labor. (2008). Available at: http://www.bls.gov/iif/oshwc/osh/os/ostb2071.pdf.

[11] Lavoie J Dunkerley CJ Kosatsky T Dufresne A. Exposure to aerosolized bacteria and fungi among collectors of commercial waste. Sci Total Environ. (2006) 370:23–8. doi: 10.1016/j.scitotenv.2006.05.016, PMID: 16930679

[12] Habtu Y Kumie A Tefera W. Magnitude and factors of occupational injury among Workers in Large Scale Metal Manufacturing Industries in Ethiopia. OALib. (2014) 1:1–10. doi: 10.4236/oalib.1101087

[13] Wahab B Ogunlola B. The nature and challenges of street sweeping in ado-Ekiti. Afr J Psychol Study Soc Iss. (2014) 7:145–67.

[14] Joy P Chitra AKJ. “A cross-sectional study to assess the health profile of street sweepers and sanitary workers in a zone of Greater Chennai Corporation, Tamil Nadu, India”. Int J Community Med Public Heal 5, no. 10. (2018) 4362–62. doi: 10.18203/2394-6040.ijcmph20183974

[15] van Kampen V Hoffmeyer F Seifert C Brüning T Bünger J. Occupational health hazards of street cleaners: a literature review considering prevention practices at the workplace. Int J Occup Med Environ Health. (2020) 33:701–32. doi: 10.13075/ijomeh.1896.01576 32939096

[16] ILO . Guidelines on the promotion of decent work and road safety. (2019) Geneva: International Labor Organization (ILO). 23–27.

[17] Degavi G Debbarma S Adola SG Safayi BL Gemeda U Utura T . Occupational hazards and its relation with health-seeking and practicing behaviors among sanitary workers in southern Ethiopia. Int. J. Africa Nurs. Sci. (2021) 15:100339.

[18] Page M McKenzie JE Bossuyt PM Boutron I Hoffmann TC Mulrow CD . The PRISMA 2020 statement: an updated guideline for reporting systematic reviews. Syst Rev. (2021) 10:10–89. doi: 10.1186/s13643-021-01626-4 33781348 PMC8008539

[19] Munn Z Moola S Lisy K Riitano D Tufanaru C. Methodological guidance for systematic reviews of observational epidemiological studies reporting prevalence and incidence data. Int J Evid Based Healthc. (2015) 13:147–53. doi: 10.1097/XEB.0000000000000054, PMID: 26317388

[20] Chandra W Arora R. Tuberculosis and other chronic morbidity profile of sewage workers of Delhi. Indian Journal Tuberc. (2019) 66:144–9. doi: 10.1016/j.ijtb.2018.09.003, PMID: 30797273

[21] Cyprowski M Sobala W Buczyńska A Szadkowska-Stańczyk I. Endotoxin exposure and changes in short-term pulmonary function among sewage workers. Int J Occup Medi Env Health. (2015) 28:803–11. doi: 10.13075/ijomeh.1896.00460, PMID: 26224492

[22] Cyprowski M Stobnicka-Kupiec A Górny R Gołofit-Szymczak M Ptak-Chmielewska A Ławniczek-Wałczyk A. Across-shift changes in upper airways after exposure to bacterial cell wall components. Annals Agri Env Medi. (2019) 26:236–41. doi: 10.26444/aaem/106112, PMID: 31232052

[23] Shadab M Agrawal DK Aslam M Islam N Ahmad Z. Occupational health hazards among sewage workers: Oxidative Stress and Deranged Lung Functions. J Clin Diagn Res. (2014) 8:BC11–2. doi: 10.7860/JCDR/2014/5925.4291 PMC4064928 24959435

[24] Heldal K Madsø L Huser PO Eduard W. Exposure, symptoms and airway inflammation among sewage workers. Ann Agric Environ Med. (2010) 17:263–8. PMID: 21186769

[25] Heldal K Barregard L Larsson P Ellingsen DG. Pneumoproteins in sewage workers exposed to sewage dust. Int Arch Occup Environ Health. (2013) 86:65–70. doi: 10.1007/s00420-012-0747-7, PMID: 22350277 PMC3535374

[26] Heldal K Austigard ÅD Svendsen KH Einarsdottir E Goffeng LO Sikkeland LI . Endotoxin and hydrogen Sulphide exposure and effects on the airways among waste water workers. Annals Work Expos Health. (2019) 63:437–47. doi: 10.1093/annweh/wxz020, PMID: 30938763

[27] Shadab M Agrawal DK Aslam M Islam N Ahmad Z. Occupational health hazards among sewage workers: oxidative stress and deranged lung functions. J Clin Diagn Res. (2014) 8:BC11. , PMID: 24959435 10.7860/JCDR/2014/5925.4291 PMC4064928

[28] Ajay KT Vatsala AR Danyakumar G Bondadae SY. A study of impairment of lung functions in adult sweepers. J Pharm Sci Res. (2014) 6:239–41.

[29] Anwar S Mehmood N Nasim N Khurshid M Khurshid B. Sweeper's lung disease: a cross-sectional study of an overlooked illness among sweepers of Pakistan. Int J Chron Obstruct Pulmon Dis. (2013) 8:193–7. doi: 10.2147/COPD.S40468, PMID: 23626464 PMC3632582

[30] Arora R Kaur H. Lung function response to dust in Safai workers. Int J Med Dent Sci. (2016) 5:1038–41. doi: 10.19056/ijmdsjssmes/2016/v5i1/83572

[31] Erah F Petra EE Omorogbe I Joy TO Efedaye O Adeniyi OB. Effect of dust on the respiratory health of street sweepers in Benin City, Edo state, Nigeria 2018: Benin, Nigeria

[32] Johncy SSST Samuel TV Jayalakshmi MK Dhanyakumar G Bondade SY. Prevalence of respiratory symptoms in female sweepers. Int J Biomed Res. (2014) 5:408–10.

[33] Stambuli P . Occupational respiratory health symptoms and associated factors among street sweepers in Ilala, municipality. (Doctoral dissertation, Muhimbili University of Health and Allied Sciences). (2012):23–38.

[34] Mohammad Afzal S Agrawal DK Ahmad Z Aslam M. A cross sectional study of pulmonary function tests in street cleaners in Aligarh, India. Biomed Res. (2013) 24:449–52.

[35] Eshaghi Sani H Department of Occupational Medicine, Faculty of Medicine Najaf Najafi M Department of Community of Medicine, Clinical Research Unit, Faculty of Medicine Sharifi H Rezident, Department of Occupational Medicine, Faculty of Medicine . Spirometry pattern and respiratory symptoms in sweepers. Majallah-i pizishkī-i hurmuzgān. (2017) 21:271–7. doi: 10.29252/hmj.21.4.271,

[36] Sangolli B Rashmi BM Jagadish S Sreeharsha CB. A cross-sectional study of pulmonary function tests among the municipal street sweepers of Chitradurga District, Karnataka. Indian J Immunol Respir Med. (2018) 3:108–13.

[37] Eneyew B Sisay T Gizeyatu A Lingerew M Keleb A Malede A . Prevalence and associated factors of acute respiratory infection among door-to-door waste collectors in Dessie City, Ethiopia. PLoS One. (2021) 16:e0251621. doi: 10.1371/journal.pone.0251621 33989364 PMC8121341

[38] Mostafa NS Abdel-Hamid MA AlBagoury LS. Work-related respiratory disorders among street sweepers in Cairo, Egypt, a comparative study. Egypt J Co Med. (2015) 33:85–97.

[39] Nku C Peters EJ Eshiet AI Oku O Osim EE. Lung function, oxygen saturation and symptoms among street sweepers in Calabar-Nigeria. Niger J Physiol Sci. (2005) 20:79–84. PMID: 17220917

[40] Johncy S G D Samuel TV K TA Bondade SY. Acute lung function response to dust in street sweepers. J Clin Diagn Res. (2013) 7:2126–9. doi: 10.7860/JCDR/2013/5818.3449, PMID: 24298455 PMC3843425

[41] Johncy S Dhanyakumar G Kanyakumari K Samuel T. Chronic exposure to dust and lung among female sweepers in India. Natl J Physiol Pharm Pharmacol. (2014) 4:15–9. doi: 10.5455/njppp.njppp.2014.4.140620131

[42] Juhi A . Pulmonary function test in street sweepers compared to general population of Hyderabad, India. Int J Sci Res. (2016) 5:332–4. doi: 10.36106/ijsr

[43] Emiru Z Gezu M Chichiabellu TY Dessalegn L Anjulo AA. Assessment of respiratory symptoms among SWM in, Ethiopia. J of Public Health and Epi. (2017) 9:189–97.

[44] Singh M Ladusingh L. Factors associated with chronic bronchitis among municipal sanitary Workers in Varanasi, India. Asian J Epidemiol. (2017) 10:101–7. doi: 10.3923/aje.2017.101.107

[45] Athanasiou M Makrynos G Dounias G. Respiratory health of municipal solid waste workers. Occup Med. (2010) 60:618–23. doi: 10.1093/occmed/kqq127, PMID: 20819804

[46] Fahim A El-Prince M. Passive smoking, pulmonary function and bronchial hyper-responsiveness among indoor sanitary workers. Ind Health. (2012) 50:516–20. doi: 10.2486/indhealth.2012-0003, PMID: 23047075

[47] Bonanni P Comodo N Pasqui R Vassalle U Farina G Lo Nostro A . Prevalence of hepatitis a virus infection in sewage plant workers of Central Italy: indicate if vaccination justified? J Vaccine. (2000) 19:844–9. doi: 10.1016/S0264-410X(00)00227-9, PMID: 11115708

[48] Divizia M Cencioni B Palombi L Panà A. Sewage workers: risk of acquiring enteric virus infections including hepatitis a virus. New Microbiol. (2008) 31:337–41. 18843887

[49] Levin M Froom P Trajber I Lahat N Askenazi S Lerman Y. Risk of hepatitis a virus infection among sewage workers in Israel. Arch Environ Health. (2000) 55:7–10. doi: 10.1080/00039890009603378, PMID: 10735513

[50] Montuori P Negrone M Cacace G Triassi M. Wastewater workers and hepatitis a virus infection. Occup Med. (2009) 59:506–8. doi: 10.1093/occmed/kqp092, PMID: 19561054

[51] Toseva EI Atanasova MV Turnovska TH. Seroprevalence of anti-HAV Total antibodies among wastewater workers. Int J Occu Medi Env Health. (2018) 31:307–15. 10.13075/ijomeh.1896.01161 29072711

[52] El-Esnawy N . Examination for hepatitis E virus in wastewater treatment plants and workers by nested RT-PCR and ELISA. J Egypt Public Health Assocc. (2000) 75:219–31. 17219857

[53] Venczel L Brown S Frumkin H Simmonds-Diaz J Deitchman S Bell BP. Prevalence of hepatitis a virus infection among sewage workers in Georgia. Am J Ind Med. (2003) 43:172–8. doi: 10.1002/ajim.10174, PMID: 12541272

[54] Hassanein F Masoud I Shehata A. Infection hazard of exposure to intestinal parasites, viruses among sewage workers. Parasitologists United Journal. (2019) 12:130–8. doi: 10.21608/puj.2019.13679.1047

[55] Van Hooste W Charlier AM Rotsaert P Bulterys S Moens G van Sprundel M. Work-related *Helicobacter pylori* infection among sewage workers in municipal wastewater treatment plants in Belgium. BMJ Journals, Occup Envi Med. (2010) 67:01–10. 10.1136/oem.2008.040436 20133459

[56] Jeffree M Mori D Yusof NA Atil AB Lukman KA Othman R . High incidence of asymptomatic leptospirosis among urban sanitation workers from Kota Kinabalu, Sabah, Malaysian Borneo. Sci Rep. (2020) 10:19442. doi: 10.1038/s41598-020-76595-0 33173153 PMC7655852

[57] Fuhrimann S Winkler MS Stalder M Niwagaba CB Babu M Kabatereine NB . Disease burden due to gastrointestinal pathogens in a wastewater system in Kampala, Uganda. Microbial Risk Analysis. (2016) 4:16–28. doi: 10.1016/j.mran.2016.11.003

[58] Schöniger-Hekele M Petermann D Weber B Müller C. *Tropheryma whipplei*in the environment: survey of sewage plant influxes and sewage plant workers. Appl Environ Microbiol. (2007) 73:2033–5. doi: 10.1128/AEM.02335-06, PMID: 17277223 PMC1828826

[59] Thorn J Beijer L. Work-related symptoms and inflammation among sewage plant operatives. Int J Occ Environ Health. (2013) 10:84–9. doi: 10.1179/oeh.2004.10.1.84 15070030

[60] Thorn J Beijer L Rylander R. Work related symptoms among sewage workers: a nationwide survey in Sweden. Occup Environ Med. (2002) 59:562–6. doi: 10.1136/oem.59.8.562, PMID: 12151615 PMC1740343

[61] Lee J Thorne PS Reynolds SJ O’Shaughnessy PT. Monitoring risks in association among wastewater treatment plant workers. J Occup Environ Med. (2007) 49:1235–48. doi: 10.1097/JOM.0b013e3181568b40, PMID: 17993928

[62] Sangkham S Thongtip S Sakunkoo P. Occupational health hazard exposure and health problems among solid waste collectors Thailand. J Pub H Dev. (2021) 19:206–218.

[63] Lenka AK In: J.N. University , editor. Health, work, and state response toward person engaged in sanitation work: some issues and challenges health, safety and well-being of Workers in the Informal Sector in India. 1st ed. India: Springer Nature Singapore Pte Ltd. (2019). 01–16.

[64] Preisser A Zhou L Garrido MV Harth V. Measured by the oxygen uptake in the field, the work of refuse collectors. Int Arch Occup Environ Health. (2016) 89:211–20. doi: 10.1007/s00420-015-1064-8, PMID: 26088744 PMC4724371

[65] Uhunamure S Edokpayi JN Shale K. Occupational health risk of waste pickers: a case study of northern region of South Africa. J Environ Public Health. (2021) 2021:1–12. doi: 10.1155/2021/5530064, PMID: 34512770 PMC8424242

[66] Douwes J McLean D Slater T Pearce N. Work-related symptoms in sewage treatment workers. Ann Agric Environ Med. (2001) 8:39–45. doi: 10.1002/ajim.1060, PMID: 11426923

[67] Krajewski J Cyprowski M Szymczak W Gruchala J. Health complaints from workplace exposure to bioaerosols: a questionnaire study in sewage workers. Ann Agric Environ Med. (2004) 11:199–204. PMID: 15627324

[68] Smit L Spaan S Heederik D. Endotoxin exposure and symptoms in wastewater treatment workers. Am J Ind Med. (2005) 48:30–9. doi: 10.1002/ajim.20176, PMID: 15940720

[69] El-Wahab EW Eassa SM Lotfi SE Kotkat AM Shatat HZ. Prevalence, immune status and factors associated among Egyptian MSW workers. J Virol Antivir Res. (2015) 4:01–4.

[70] Giri P Kasbe AM Aras R. Occupational stress among sewage workers. Biomedicine. (2011) 31:372–7.

[71] Lenka AK . “Health, Work, and State Response Toward Person Engaged in Sanitation Work: Some Issues and Challenges”. Health, Safety and Well-Being of Workers in the Informal Sector in India: Lessons for Emerging Economies. (2019):215–231.

[72] El-Wahab EWA Eassa SM Lotfi SE Kotkat AM Shatat HZ. Seroprevalence, immunostatus and factors associated with blood borne viral infections among Egyptian municipal solid waste workers. J Virol Antivir Res. (2015) 4:2.

