# Supplementary material for: Global systematic review of occupational health and safety outcomes among sanitation and hygiene workers
Source: Front Public Health. 2023 Dec 19;11:1304977. doi: 10.3389/fpubh.2023.1304977 (PMC10763668; doi:10.3389/fpubh.2023.1304977)
Supplement: Supplementary file 1 [file Data_Sheet_1.docx]

**Supplementary Material**

**World wide**

Of 23 countries, 14 (61%) countries were from developed countries and 9 (39%) of them were from developing countries (Supplementary Figure 1).

Sup Figure 1 Identified Studies from developing and developed

**Studies from the Countries**

From a total of 51 studies majority (15 studies) of them were identified from India. Then followed by Egypt, Italy and Norway equally shared 3 studies (Sup Figure 2).

Sup Figure 2 Number of Studies identified Across the world

**Statistical Technique**

More than half percent of the studies used logistic regression, binary and multiple, univariate, bivariate and multivariate regression analysis. Followed by chi-square with other models such as chi square with fisher’s exact test, logistic regression, multiple comparison and binary logistic regression accounted 38%. The third one is descriptive reports such as frequency, mean and standard deviation accounted 8% (Supplementary Figure 3).

Sup. Figure 3 Statistical used in Reviewed studies on Occupational health and safety outcomes, 2022

**Study Design**

Moreover, majority of the studies used cross sectional studies design with structured, standard questionnaires alone, questionnaires with observational checklist. A few of them used questionnaires with spirometer, blood test and stool examination. As Table showed stool examination demonstrated for gastroenteritis to detect the presence of for microbial, intestinal parasite infections, hepatitis A and B virus in sanitary workers, while spirometer measurement used for respiratory examination (Table 1)**.** Beside, in sewage workers, waste water was analyzed to know the load of bacteriology in sewage water and waste treatment either risk or not if the possibilities of exposure (Sup. Figure 4).

Sup. Figure 4: Assessment tools used for assessment of occupational Health and safety outcomes

**Common findings for sanitary workers**

From the current review, for all categories of sanitary workers it can summarize the common assessment tools and outcomes as follow. For instance, in municipality solid waste collectors almost all studies used standard questionnaires for assessment tool while outcomes like musculoskeletal disorders/MSDs/ and parasitic intimal infection were common for all studies (Supplementary Table 1)

Sup. Table 1 Most common for type of sanitary workers in tool and outcomes

| **S.no** | **Sanitary workers** | **Most common used** | **Most common Outcomes** |
| --- | --- | --- | --- |
|  | Municipality Solid waste Collectors | - Questionnaires | - MSDs - Intestinal parasites |
|  | Sewage and waste treatment workers | - Blood sample - Stool sample | - Hepatitis B virus - Intestinal parasites |
|  | Street Sweepers | - Questionnaires - Spirometer measurement | - Respiratory problems like Acute respiratory Infection, impairs lung function, cough, chest pain , sneezing |
|  | Health care facilities cleaners | - Standard questionnaires | - Sharp injuries - Needle injuries - Hepatitis B virus |
|  | General sanitary workers | - Nordic Questionnaires - Spirometer measurement - Stool sample | - Cardiovascular - Musculoskeletal Disorders/MSDs - Infections, - Skin problems - Respiratory problems - Gastroenteritis |
|  | Street sweepers and waste collectors | - Questionnaires - Spirometer measurement | - Musculoskeletal Disorders/MSDs - Acute respiratory Infection - impairs lung function - Cough, chest pain , sneezing |

Supplementary Table 2 Overall studies result by nine statement of JBI

| **Statement of JBI for Identified Studies (n=51)** | **Total Yes (Yes =X)** | **%** |
| --- | --- | --- |
| 1. Was the sample frame appropriate to address the target population? | 34 | 66.67% |
| 1. Were study participants sampled in an appropriate way? | 24 | 46.67% |
| 1. Was the sample size adequate? | 44 | 86.67% |
| 1. Were the study subjects and the setting described in detail? | 43 | 84.44% |
| 1. Was the data analysis conducted with sufficient coverage of the identified sample? | 39 | 75.56% |
| 1. Were valid methods used for the identification of the condition? | 36 | 71.11% |
| 1. Was the condition measured in a standard, reliable way for all participants? | 41 | 81.11% |
| 1. Was there appropriate statistical analysis? | 44 | 86.67% |
| 1. Was the response rate adequate, and if not, was the low response rate managed appropriately? | 48 | 94.44% |
| **Overall evaluation** | 354 | **77%** |
